# BSE-YOLO: An Enhanced Lightweight Multi-Scale Underwater Object Detection Model

**DOI:** 10.3390/s25133890

**Published:** 2025-06-22

**Authors:** Yuhang Wang, Hua Ye, Xin Shu

**Affiliations:** School of Computer Science, Jiangsu University of Science and Technology, Zhenjiang 212100, China; 222210712227@stu.just.edu.cn (Y.W.); jsjxy_yh@just.edu.cn (H.Y.)

**Keywords:** underwater object detection, YOLOv10n, bidirectional feature pyramid network, multi-scale attention synergy, lightweight network

## Abstract

Underwater images often exhibit characteristics such as low contrast, blurred and small targets, object clustering, and considerable variations in object morphology. Traditional detection methods tend to be susceptible to omission and false positives under these circumstances. Furthermore, owing to the constrained memory and limited computing power of underwater robots, there is a significant demand for lightweight models in underwater object detection tasks. Therefore, we propose an enhanced lightweight YOLOv10n-based model, BSE-YOLO. Firstly, we replace the original neck with an improved Bidirectional Feature Pyramid Network (Bi-FPN) to reduce parameters. Secondly, we propose a Multi-Scale Attention Synergy Module (MASM) to enhance the model’s perception of difficult features and make it focus on the important regions. Finally, we integrate Efficient Multi-Scale Attention (EMA) into the backbone and neck to improve feature extraction and fusion. The experiment results demonstrate that the proposed BSE-YOLO reaches 83.7% *mAP*@0.5 on URPC2020 and 83.9% *mAP*@0.5 on DUO, with the parameters reducing 2.47 M. Compared to the baseline model YOLOv10n, our BSE-YOLO improves *mAP*@0.5 by 2.2% and 3.0%, respectively, while reducing the number of parameters by approximately 0.2 M. The BSE-YOLO achieves a good balance between accuracy and lightweight, providing an effective solution for underwater object detection.

## 1. Introduction

**Figure 1:**
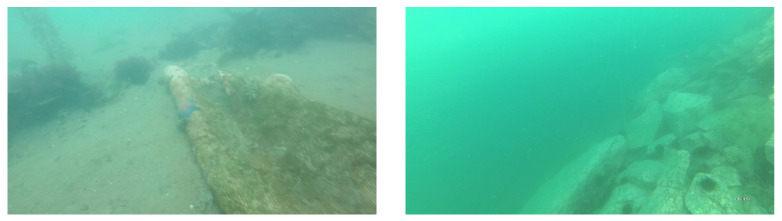
The underwater images have characteristics such as low contrast, noise and color distortion.

The ocean covers 71% of Earth’s surface, playing a crucial role in global climate, species survival, and ecological balance, and serving as an essential resource for human civilization. Underwater object detection represents a challenging yet promising field within computer vision, offering significant applications in marine environmental monitoring, ecological preservation, and resource exploration. Recent advancements in autonomous underwater vehicles (AUVs) and unmanned underwater vehicles (UUVs) have substantially enhanced the availability and quality of underwater images [1,2]. However, as shown in Figure 1, due to the effects of light attenuation and scattering, underwater images are plagued by issues such as low contrast, noise, and color distortion [3,4].

In this study, the primary underwater object detection challenges we aim to address encompass the following: (1) significant variations in shape and size, as well as issues with blurred, minute objects and clustered objects; (2) low contrast, which makes it hard to distinguish objects from their backgrounds. These factors severely degrade the performance of detection models and lead to false detections and missed targets. Traditional object detection methods often fail to achieve the accuracy and speed required by modern applications when confronted with these issues. Furthermore, although this study mainly optimizes the problems of low contrast and detection difficulties such as small and blurred objects, degradation factors such as noise and color distortion have not been completely solved and remain important challenges in the current field of underwater target detection.

In recent years, deep learning technologies have developed rapidly and been widely used in different fields such as defect detection, underwater image enhancement, and object recognition [5,6,7,8]. The object detection algorithms based on deep learning are mainly divided into two categories: one-stage methods and two-stage methods. One-stage algorithms, such as the You Only Look Once (YOLO) series [9,10,11,12,13] and SSD [14], directly classify and locate objects in the input image, thus offering faster detection speed. Two-stage algorithms, such as R-CNN [15], Fast-RCNN [16], and Faster-RCNN [17], initially generate candidate regions and subsequently perform classification and localization refinement on these regions. Compared to one-stage methods, they achieve higher detection accuracy, albeit with a reduction in processing speed.

Currently, most research efforts concentrate on enhancing the detection accuracy of models, but at the same time, they have also increased the model parameters and computational complexity. In addition, underwater equipment usually has limited storage capacity and processing power. Therefore, when improving the detection accuracy, it is also necessary to consider the parameters and computational complexity to achieve an effective balance between efficiency and lightweight.

This paper introduces a lightweight underwater object detection model BSE-YOLO, which integrates the improved Bidirectional Feature Pyramid Network (BiFPN) [18], a modified C2f structure named Synergistic C2f (S_C2f), where the proposed Multi-Scale Attention Synergy Module (MASM) is applied, and the Efficient Multi-Scale Attention (EMA) module [19]. The proposed BSE-YOLO mainly aims to improve the perceptibility of difficult samples and small, blurred, and clustered objects to enhance the model’s detection accuracy and multi-scale robustness. Secondly, BSE-YOLO tries to reduce or not change the parameters and computational complexity to achieve a balance between accuracy and light weight. The main contributions of this paper are summarized as follows:

(1) We incorporate an improved Bi-FPN to replace the original neck of YOLOv10n, achieving a reduction in parameters and computational costs while improving the model’s feature fusion capabilities.

(2) We propose a MASM and apply it to the bottleneck of C2f to generate a modified C2f structure named Synergistic C2f (S_C2f), which can dynamically adjust feature maps, improve the focus on critical regions, and enhance the spatial feature extraction and fusion through depthwise separable convolutions.

(3) We integrate the EMA in the backbone and neck to strengthen the focus on multi-scale features and further improve detection accuracy.

The rest of this paper is organized as follows: Section 2 reviews the related work. Section 3 provides a detailed description of the proposed model. Section 4 presents experimental results and discussions. The conclusions are drawn in Section 5.

## 2. Related Work

### 2.1. Underwater Object Detection

Due to factors such as low contrast, noise, target aggregation, blurriness, small size, and complex morphological variations of underwater objects, traditional object detection algorithms typically exhibit performance degradation when applied to underwater images. Meanwhile, the rapid advancement of deep learning has led to its gradual replacement of traditional algorithms. Deep learning offers effective solutions for underwater object detection. Chen et al. [20] proposed a Sample-WeIghted hyPEr Network (SWIPENET), which significantly enhances the detection capability for small targets by generating multiple high-resolution and semantically rich feature maps, thereby effectively addressing the challenges of blurriness and noise in underwater images. Liu et al. [21] proposed an underwater target detection algorithm based on Faster R-CNN to address common issues such as low contrast and target blurriness, reducing missed detections and false alarms, and improving detection accuracy and efficiency. Pan et al. [22] proposed an improved multi-scale residual neural network (M-ResNet), which enhances the accuracy of detecting underwater objects of different sizes with multi-scale operations. Gao et al. [23] designed a detection module with flexible and adaptive point representation and a new weighted loss function in the developed PE-Transformer. This approach enables the acquisition of semantic details of small-scale underwater targets in complex environments, improving the detection accuracy of underwater targets while encouraging convergence of the network.

In addition, the YOLO series maintains a relatively fast detection speed while achieving a high detection accuracy. Cai et al. [24] proposed a novel underwater detection method that simultaneously trains two deep learning detectors based on YOLOv5 within a weakly supervised learning framework. The detectors mutually teach each other by selecting clearer samples observed during training. Zhang et al. [25] proposed an enhanced lightweight YOLOv4-Lite model by integrating MobileNetv2, depthwise separable convolutions, and an improved Attention Feature Fusion Module (AFFM). This approach significantly reduces computational overhead during training while preserving high detection accuracy, thereby achieving an optimal balance between precision and inference speed. Liu et al. [26] developed the TC-YOLO network based on YOLOv5s by incorporating self-attention and coordinated attention mechanisms from transformers into both the backbone and neck of the network. Additionally, they proposed an optimal transport label assignment strategy to enhance feature extraction and improve the utilization efficiency of training data. Feng et al. [27] designed the CEH-YOLO by integrating high-order deformable attention (HDA), enhanced spatial pyramid fast pooling (ESPPF), and customized composite detection (CD) modules, achieving remarkable performance in underwater small object detection. The above research primarily concentrated on enhancing the detection accuracy of the model in complex scenarios, with less emphasis on making the model lightweight. In other words, there remains room for further optimization between detection efficiency and accuracy.

### 2.2. Attention Mechanism

The human visual system can naturally identify salient regions in complex scenes. By emulating this capability, the attention mechanism dynamically adjusts weights based on input image features [28], thereby enabling the prioritization of critical information processing with limited computational resources. In underwater detection tasks, objects are typically small and dense. Several researchers have applied attention mechanisms to existing networks and found that these mechanisms can significantly enhance the detection accuracy of underwater objects. Wang et al. [29] integrated Channel and Spatial Fusion Attention (CSFA) into the YOLOv5. This approach not only maintains focus on spatial information but also enhances the extraction of key features. Yan et al. [30] incorporated the CBAM attention into YOLOv7. By weighting and enhancing features in both spatial and channel dimensions, this approach captures local correlations of feature information, refines the model’s focus on feature details, and consequently improves detection accuracy.

According to the dimensions they focus on and the features they emphasize, attention mechanisms are typically categorized into three types: spatial attention, channel attention, and hybrid attention. Spatial attention enables the model to focus on spatial positions in the image that are most relevant to the current task. For example, Spatial Transformer Networks (STNs) [31] can transform deformed data in the spatial domain and automatically capture salient features from critical regions. Channel attention assigns different weights to each channel to reflect their varying importance. This enables the model to filter out the most relevant features for the current task from a multitude of features, thereby enhancing the model’s efficiency in feature utilization. Squeeze-and-Excitation Networks (SENet) [32] exemplify channel attention. The Convolutional Block Attention Module (CBAM) [33], as mentioned in the previous study, is a classical hybrid attention mechanism. It integrates spatial and channel attention mechanisms sequentially and has been extensively utilized in convolutional neural network architectures. In addition to the aforementioned traditional attention mechanisms, there is a more granular pixel attention mechanism that focuses on each pixel of the image. By assigning a weight to each pixel, this approach enables the model to concentrate on key information within the image while disregarding irrelevant backgrounds or noise. For example, the pixel-wise contextual attention network (PiCANet) proposed by Liu et al. [34] generates an attention map for each pixel, where each attention weight reflects the contextual relevance at the corresponding spatial position. Currently, several researchers are actively exploring the synergistic effects between different types of attention mechanisms to leverage their respective advantages and enhance performance in visual tasks. Si et al. [35] proposed a novel spatial–channel synergistic attention module (SCSA) by investigating the synergy between spatial and channel attention.

Inspired by [35], we propose a Multi-Scale Attention Synergy Module (MASM), which integrates channel, spatial, and pixel attention. MASM is utilized to develop the S_C2f structure. Furthermore, we propose BSE-YOLO, a model that combines BiFPN, S_C2f, and EMA. Compared with YOLOv10n, our BSE-YOLO achieves higher detection accuracy for underwater objects while reducing parameters.

## 3. Methods

### 3.1. The Architecture of YOLOv10n

You Only Look Once (YOLO) is a representative one-stage detector with both detection speed and accuracy. It reformulates object detection as a regression task and directly predicts object categories and locations. YOLOv10 [36] is the new series of real-time, end-to-end YOLO detectors, comprising six variants: YOLOv10n, YOLOv10s, YOLOv10m, YOLOv10b, YOLOv10l and YOLOv10x. We select YOLOv10n, which has the fewest parameters, as the baseline and further optimize it to be more lightweight.

**Figure 2:**
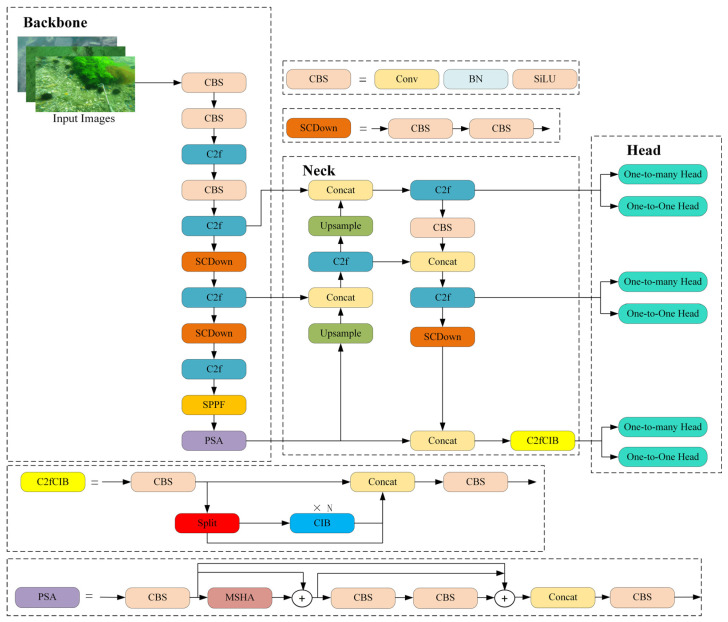
The original architecture of YOLOv10n.

As shown in Figure 2, the structure of YOLOv10n is divided into three parts: backbone, neck, and head. The backbone extracts multi-scale features from the input image and mainly consists of CBS modules, SCDown modules, C2f modules, the fast spatial pyramid pooling (SPPF) modules, and partial self-attention (PSA). Each CBS block is composed of a convolution (Conv) layer, a batch normalization layer (BN), and a SiLU activation function. The SCDown module is made up of two convolution (Conv) layers, where the first convolution adjusts the number of channels and the other is used for downsampling. The C2f module consists of two convolution layers and some bottlenecks. The SPPF module performs max pooling on the input feature maps using pooling kernels of sizes 5 × 5, 9 × 9, and 13 × 13. It then aggregates the pooled feature maps via a concatenation layer, which captures multi-scale features. The PSA module consists of four convolution layers and multi-head self-attention (MHSA). The neck fuses and enhances the multi-scale features extracted by the backbone. It leverages the Feature Pyramid Network (FPN) and Path Aggregation Network (PAN) to integrate semantic and spatial information from multiple feature maps, thereby improving detection performance for various targets. The fused feature maps are subsequently provided to the detection head. The primary function of the head is to predict object localization and classification.

### 3.2. The Structure of the Proposed BSE-YOLO

**Figure 3:**
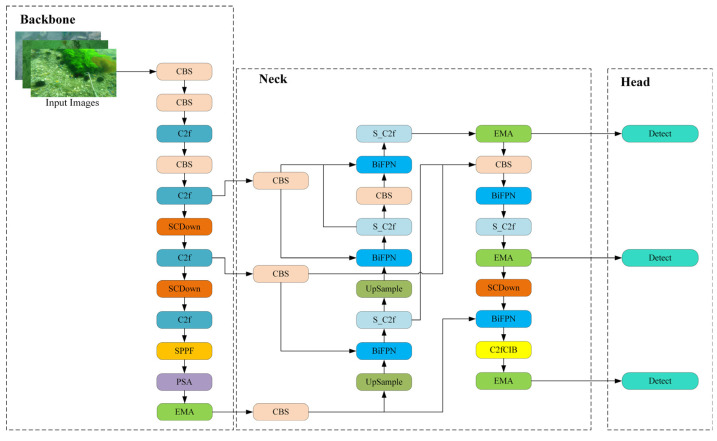
The framework of the proposed BSE-YOLO.

To enhance the detection accuracy and make the architecture more lightweight, we propose BSE-YOLO. The structure of BSE-YOLO is illustrated in Figure 3. Firstly, BiFPN is introduced to optimize the original Feature Pyramid Network to improve multi-scale feature fusion and detection performance without incurring substantial additional computational costs. Additionally, an improved S_C2f structure replaces the corresponding module in the neck to enhance the model’s ability to handle complex samples with low contrast and morphological variations. Furthermore, EMA is integrated into both the backbone and neck to strengthen the model’s focus on multi-scale features and further improve feature extraction and fusion capabilities.

#### 3.2.1. Improved Bidirectional Feature Pyramid Network (BiFPN)

The Feature Pyramid Network (FPN) [37], which was first introduced in YOLOv3, employs a bottom-up feature extraction path to generate a pyramid-shaped feature map by leveraging features from different layers of a convolutional neural network. Subsequently, through a top-down feature fusion path, it progressively refines the details of high-level feature maps to enrich the semantic depth of the network. However, during the feature upsampling from high-level to low-level, spatial details from low-level features are susceptible to loss. To address this issue, the subsequent YOLOv4 network introduced the Path Aggregation Network (PAN) [38]. By enhancing the bottom-up path, PAN enables each layer’s feature maps to effectively acquire spatial details from lower layers, thereby complementing the FPN framework. This improvement strengthens the feature representation of low-level features and improves the model’s learning capability and accurate target localization performance. Subsequently, feature fusion networks, which integrate FPN with PAN, further optimize the model’s detection performance.

**Figure 4:**
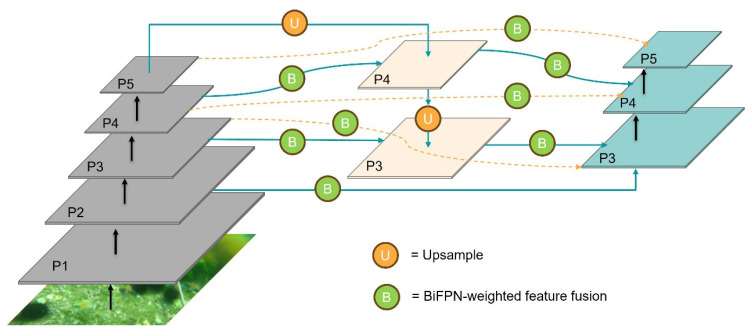
The structure of the optimized Feature Pyramid Network. “U” = upsample, “B” = BiFPN-weighted feature fusion.

Although the combined application of FPN and PAN has achieved significant breakthroughs in object detection, there remains room for improvement in detecting small objects. Given that most underwater objects are relatively small, their features are prone to loss during the progressive downsampling process in deep neural networks. To address this issue, we introduce an improved BiFPN for optimization, as illustrated in Figure 4. BiFPN establishes bidirectional connections between top-down and bottom-up paths, enabling more efficient information exchange and fusion of features across different scales. Additionally, it adopts a weighted fusion mechanism to replace the simple feature summation used in traditional Feature Pyramid Networks. BiFPN improves model accuracy and stability by assigning learnable weights to each input feature map, allowing the network to adoptedly emphasize more informative features and suppress less relevant ones during the feature fusion process. This process is described by the following formula:

(1) $$O=\sum_{i}\frac{w_{i}\cdot I_{i}}{\epsilon +\sum_{i}w_{i}}$$

where O denotes the result after fusion; $w_{i}$ represents the weight of the input feature $I_{i}$; a small constant ϵ is set to 0.0001 to ensure that the computation remains robust when the sum of weights becomes very small or zero, which could lead to division by zero or amplified errors.

In addition, single input nodes that contribute minimally to effective feature fusion are removed. The feature map P2 is integrated into the original BiFPN, and skip connections are established between the original input and output nodes. By applying weighted feature fusion, the network’s ability to fuse multi-scale features is enhanced while improving efficiency.

#### 3.2.2. Multi-Scale Attention Synergy Module (MASM)

Images captured in underwater environments often exhibit low contrast and significant variations in object shapes. Traditional convolutional networks are prone to losing details in these images and are insensitive to object deformations, resulting in inadequate adaptability to complex samples, and may overlook critical changes, leading to missed detections and false alarms. Therefore, we propose a Multi-Scale Attention Synergy Module (MASM), which enhances the model’s focus on important regions of the image by combining attention from different scales and improves the spatial feature extraction and fusion ability through depthwise separable convolutions. This module aims to improve the perception of difficult features in samples.

**Figure 5:**
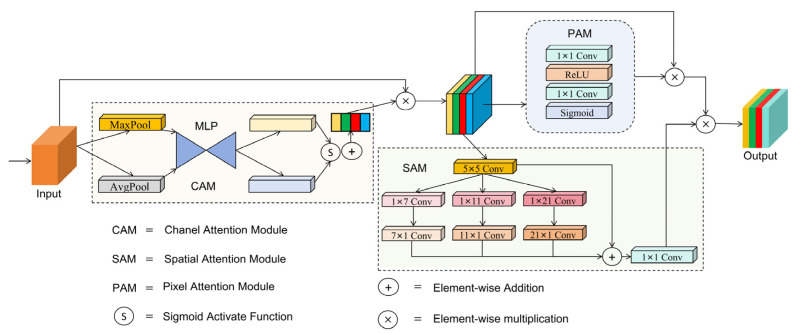
The overall architecture of MASM.

The framework of this module is shown in Figure 5. By integrating channel attention, spatial attention and pixel attention, we establish the synergy mechanism to enhance feature representation. Firstly, the input features are weighted through the channel attention module to obtain enhanced channel features. Subsequently, the spatial attention module processes the generated features to refine the spatial details, while the pixel attention module adjusts these features at the pixel level. Finally, the output is a weighted combination of spatial and pixel attention outputs, which improves the model’s ability to capture both global and local important features.

Specifically, the channel attention module first applies adaptive pooling to the input feature maps along the channel dimension to compute the results of both average pooling and max pooling. Subsequently, the pooled feature maps are passed through two 1 × 1 convolutional layers followed by the *ReLU* activation function and then normalized by the Sigmoid activation function. Finally, the channel attention map, which determines the importance of each channel, is generated by the weighted sum of the average pooling and max pooling results. The channel attention module is described in Formulas (2)–(4), where $X_{Input}$ represents the input feature map; AvgPool(⋅) and MaxPool(⋅) are the average pooling and max pooling operations applied to $X_{Input}$; ${Conv}_{k=1}$ represents a 1 × 1 convolution layer; σ(⋅) denotes the Sigmoid activation function; $fc_{avg}$ and $fc_{max}$ denote the intermediate feature representations; and $C_{attention}$ denotes the final channel attention map.

(2) $$fc_{avg}={Conv}_{k=1}(ReLU({Conv}_{k=1}(AvgPool(X_{Input}))))$$

(3) $$fc_{max}={Conv}_{k=1}(ReLU({Conv}_{k=1}(MaxPool(X_{Input}))))$$

(4) $$C_{attention}=\sigma (fc_{avg}\;+fc_{max})$$

Then, the generated channel attention map is element-wise multiplied with the input feature map to generate the weighted feature map. The result is input to both the pixel and spatial attention modules. In the pixel attention module, a pixel attention map is generated through two 1 × 1 convolutional layers and *ReLU* and Sigmoid activation functions. The original input is then multiplied by the pixel attention map to highlight important pixel regions, resulting in the weighted pixel attention map. This process is described in Formulas (5)–(6), where X denotes the feature map obtained by element-wise multiplication between the input feature map $X_{Input}$ and the channel attention map $C_{attention}$; $P_{attention}$ represents the final pixel attention map.

(5) $$X=X_{Input}*C_{attention}$$

(6) $$P_{attention}=X*\sigma (Conv_{k=1}(ReLU(Conv_{k=1}(X))))$$

The spatial attention module applies depthwise separable convolutions with kernels of 5 × 5, 1 × 7, 7 × 1, 1 × 11, 11 × 1, 1 × 21, and 21 × 1 to the received weighted feature map to capture multi-scale spatial information. Then the results are summed and fused via a 1 × 1 convolution to generate the spatial attention map. Finally, this attention map is element-wise multiplied by the weighted pixel attention map to generate the final output. This process is described in Formulas (7)–(9):

(7) $$DWConv_{K}\left(X\right)=Conv_{\left(K,1\right)}(Conv_{\left(1,K\right)}({Conv}_{k=5}(X)))$$

(8) $$S_{attention}=Conv_{k=1}(\sum_{K}DWConv_{K}\left(X\right)+Conv_{k=5}(X))$$

(9) $$X_{output}=P_{attention}*S_{attention}$$

where K∈{7,11,21} is a set of kernel sizes designed to extract spatial features at different receptive fields; $DWConv_{K}\left(X\right)$ denotes depthwise separable convolution with composite kernels of 5 × 5, 1 × K, K × 1; $S_{attention}$ is the spatial attention map; $X_{output}$ is the final enhanced feature representation of element-wise multiplication between $P_{attention}$ and $S_{attention}$.

**Figure 6:**
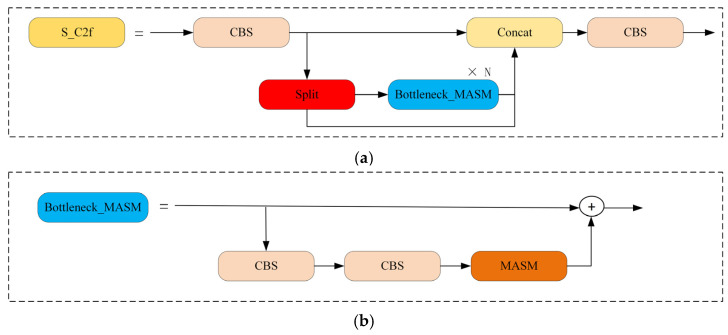
The structure of S_C2f and Bottleneck_MASM. (**a**) S_C2f; (**b**) Bottleneck_MASM.

The channel attention module helps the network learn channel importance by adaptively weighting features along the channel dimension, thereby reducing the impact of redundant information. The spatial attention module enables the network to focus on important regions in the image by learning the significance of each spatial position while suppressing irrelevant areas. Pixel attention refines features at the pixel level, which is crucial for enhancing fine-grained features. The MASM, which integrates multi-scale attention mechanisms, dynamically adjusts the feature maps in underwater object detection, thereby improving the performance of the network. In addition, we apply the MASM to the bottleneck in the C2f of the neck to generate a synergistic C2f structure named S_C2f. This structure, which combines the characteristics of MASM, can efficiently handle the complex sample features, and improve the feature fusion of the neck. The S_C2f and its bottleneck are shown in Figure 6a,b.

#### 3.2.3. Efficient Multi-Scale Attention (EMA)

Underwater environments are characterized by complex backgrounds, where target objects are typically small and tend to appear in dense clusters. Applying the attention enhances the network’s focus on key features while suppressing irrelevant information, thereby improving localization accuracy. This approach effectively boosts the network’s performance on small objects and dense targets when addressing these challenges. We integrate the EMA, which performs well in small object detection, into the backbone and neck of the YOLOv10n, aiming to improve the network’s focus on key information of multi-scale features, and further optimize the feature extraction and fusion.

**Figure 7:**
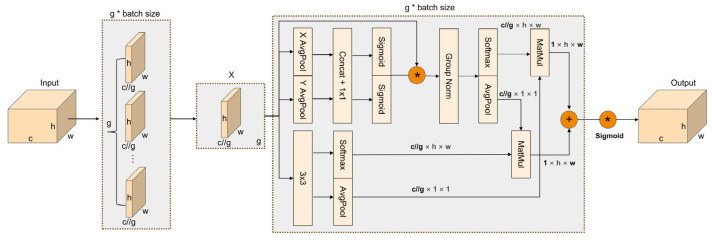
The structure of EMA. “g” = divided groups; “X AvgPool” = 1D horizontal global pooling; “Y AvgPool” = 1D vertical global pooling.

The EMA module is shown in Figure 7. This module reshapes a portion of the input feature channels to the batch dimension and transforms the channel dimension into multiple sub-features through grouping. This process ensures that spatial semantic information is evenly distributed across each feature group. Then, these features are transferred to the multi-scale parallel subnetwork to establish and process the short-term and long-term dependencies. Firstly, context information is integrated into the intermediate feature map by parallelly adopting two 1 × 1 convolution branches for horizontal and vertical 1D global average pooling and a 3 × 3 convolution branch for feature extraction, which strengthens the network’s ability to process multi-scale and multi-dimensional features. Then, the results of the two 1 × 1 convolution branches are concatenated, transformed by the Sigmoid function for feature conversion, and connected through element-wise multiplication to achieve cross-channel attention interaction. Subsequently, the outputs of the aforementioned branches perform 2D global average pooling to encode the global spatial information and apply the Softmax function for linear transformation. Finally, the ultimate spatial attention map, which collects spatial information of multi-scale features, is generated by merging the results of various branches through point-by-point multiplication. The processes of 1D global average pooling in the horizontal and vertical dimensions and the 2D global average pooling are shown in Formulas (10)–(12). $X_{c}$ indicates the input feature maps at *c*-th channel, and H and W refer to the height and width of the feature map, respectively.

(10) $$z_{c}^{H}\left(H\right)=\frac{1}{W}\sum_{0\leq i\leq W}X_{c}(H,i)$$

(11) $$z_{c}^{W}\left(W\right)=\frac{1}{H}\sum_{0\leq j\leq H}X_{c}(j,W)$$

(12) $$z_{c}=\frac{1}{H\times W}\sum_{j}^{H}\sum_{i}^{W}X_{c}(i,j)$$

## 4. Experiment and Results

### 4.1. Datasets

We use the URPC2020 and DUO datasets [39] to validate the proposed model. The URPC2020 dataset contains 5543 real underwater optical images, which encompass four common underwater objects: holothurian, echinus, scallops, and starfish. In our experiments, we partition this dataset into training, validation, and test sets in a 7:1:2 ratio, resulting in 3880 images for training, 554 images for validation, and 1109 images for testing. This division ensures adequate samples for robust model training, allows the validation set to assess model performance and prevent overfitting, and enables a more objective final evaluation in the test set.

The DUO dataset is a collection derived from recent URPC competitions [40], which has been deduplicated and relabeled. Zhao et al. [39] reorganized and shared this dataset, resulting in a total of 5208 images. This dataset contains four categories of underwater objects: holothurian, echinus, scallops, and starfish. The dataset is partitioned into training, validation, and test sets in a 7:1:2 ratio, comprising 3645 training images, 520 validation images, and 1043 test images.

The above two datasets comprehensively reflect the complexity of the real underwater environment, including low contrast that makes it difficult to distinguish objects from the background, small targets with varying degrees of blurriness, significant changes in object shape and size, and the aggregation of objects. These factors significantly increase the difficulty of the detection task and also help improve the model’s generalization ability in complex scenarios.

### 4.2. Experimental Setup

Pytorch 1.12.1 was used as the deep learning framework for all experiments. The experimental environment configuration used included an AMD Ryzen 9 5900X CPU with 32 GB of memory, NVIDIA GeForce RTX 3090 (CUDA11.3) with 24 GB of memory, and Ubuntu 16.04 with Python 3.9.16. The model training lasted for 300 epochs, the input images were uniformly adjusted to a standardized size of 640 × 640 pixels, and the Mosaic data augmentation technology was applied to enhance the robustness of the model. The batch size was set to 16, and the momentum was set to 0.937. The initial and final learning rates were fixed at 0.01, and the weight decay coefficient was set to 0.0005 to avoid overfitting. The stochastic gradient descent (SGD) was used to optimize the model.

### 4.3. Evaluation Metrics

To comprehensively evaluate the performance of the proposed model on underwater object detection, we use precision (*P*), recall (*R*), mean average precision (*mAP*), parameters (Params), giga floating-point operations per second (GFLOPs), and frames per second (FPS) as the evaluation metrics. Params and GFLOPs serve as measures for the computational complexity of the model. Meanwhile, *mAP* is employed to assess the model’s detection accuracy by taking both precision and recall into account. Furthermore, FPS is utilized to evaluate the detection speed. These indicators are calculated in the following formulas:

(13) $$Precision=\frac{TP}{TP+FP}$$

(14) $$Recall=\frac{TP}{TP+FN}$$

(15) $$mAP=\frac{\sum_{i=1}^{c}\int_{0}^{1}P\left(R\right)dR\;}{c}$$

In Equations (13) and (14), true positive (*TP*) refers to instances where the model correctly predicts positive samples as positive. False positive (*FP*) indicates cases where the model incorrectly classifies negative samples as positive. False negative (*FN*) denotes situations where the model incorrectly classifies positive samples as negative. Precision is the proportion of actual positive samples among all samples predicted as positive. Recall is the proportion of actual positive samples that are correctly predicted as positive out of all actual positive samples. In Equation (15), c denotes the number of categories, and i is the index for each class. P and R denote precision and recall, respectively. $\int_{0}^{1}P\left(R\right)dR$ computes the average precision (AP) of the i-th class.

### 4.4. Comparisons with Other Methods on URPC2020

We compared our model with some state-of-the-art models on the URPC2020 dataset. In our experiments, we employed *mAP*@0.5, *mAP*@0.5:0.95, parameters, and GFLOPs as evaluation metrics to quantify the model’s detection accuracy, size, and computational efficiency. The experimental results for SSD, YOLOv3, YOLOv4, YOLOv5s, YOLOvX [41], YOLOv8n, and FEB-YOLO were sourced from [39]. The results of YOLOv10n, RT-DETR [42] and BSE-YOLO were conducted using the same dataset and training methodology.

As illustrated in Table 1, the proposed BSE-YOLO model achieved a *mAP*@0.5 of 83.7% and a *mAP*@0.5:0.95 of 48.6%. This represents a significant improvement over SSD, with a 7.5% increase in *mAP*@0.5 and an 11.1% increase in *mAP*@0.5:0.95. Furthermore, compared to YOLOv3, YOLOv4, and YOLOvX, BSE-YOLO demonstrated superior performance, achieving improvements of 10.8%, 8.3%, and 4.0% in *mAP*@0.5, respectively. Compared with YOLOv8n and the baseline model YOLOv10n, our BSE-YOLO achieved improvements of 1.5% *mAP*@0.5 and 2.2% *mAP*@0.5, respectively. Additionally, relative to the baseline model YOLOv10n, the parameters of BSE-YOLO decreased by approximately 0.2 M. When compared with FEB-YOLO, our model attained the highest *mAP*@0.5 score of 83.7%. Furthermore, while our model exhibits more parameters and computational requirements than FEB-YOLO, its lightweight design ranks second only to FEB-YOLO. In summary, our model successfully enhances detection accuracy without significantly increasing computational demands, demonstrating both high performance and practicality.

### 4.5. Comparisons with Other Methods on DUO

To further validate the generalization capability of BSE-YOLO, we conducted comparative experiments on the enhanced DUO dataset using the same evaluation metrics. The result of LUW-DETR was obtained from [43], and the experimental results for SSD [14], Faster R-CNN [17], YOLOv3, YOLOv4, YOLOv7-Tiny, YOLOv8n, and FEB-YOLO are from [39], while the results of YOLOv10n, RTDETR [42] and BSE-YOLO were obtained under the same dataset and training settings.

As shown in Table 2, our BSE-YOLO achieved the highest *mAP*@0.5 of 83.9% and *mAP*@0.5:0.95 of 64.2%. Compared with Faster R-CNN and YOLOv7-Tiny, BSE-YOLO’s *mAP*@0.5 is 9.5% and 2.9% higher, respectively. Additionally, relative to the baseline model YOLOv10n, BSE-YOLO demonstrated significant improvements of 3.0% in *mAP*@0.5 and 2.5% in *mAP*@0.5:0.95. Although BSE-YOLO has more parameters and higher computational consumption compared to FEB-YOLO, it still achieved a 1.0% improvement in both *mAP*@0.5 and *mAP*@0.5:0.95. These results indicate that the proposed BSE-YOLO exhibits superior robustness and detection accuracy, making it effective for underwater object detection tasks.

**Figure 8:**
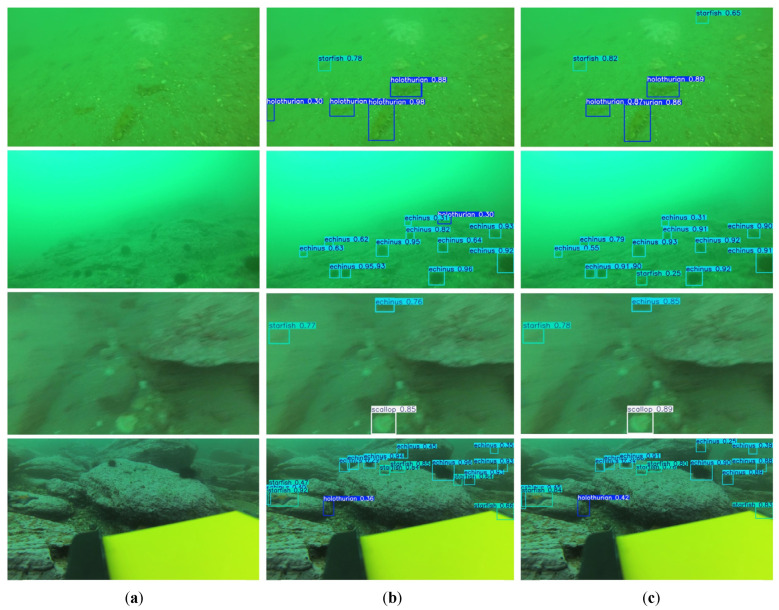
Detection results on DUO. (**a**) Raw image; (**b**) YOLOv10n; (**c**) BSE-YOLO.

To further verify the performance of BSE-YOLO, we selected several images from the test set of the DUO dataset for qualitative testing and compared them with the baseline model YOLOv10n. As shown in Figure 8, BSE-YOLO demonstrated higher detection accuracy in low-contrast images, scenes with dense small objects, and edge object detection. This indicated that BSE-YOLO had a stronger perception ability for challenging samples, thereby effectively improving its detection accuracy in complex underwater scenes.

### 4.6. Ablation Study

In this section, we conducted ablation studies on the URPC2020 dataset to further validate the effectiveness of the improvement modules. All experiments are performed under the same conditions without utilizing pre-trained weights. We use *mAP*@0.5, *mAP*@0.5:0.95, parameter count, GFLOPs, and FPS as evaluation metrics.

As shown in Table 3, we selected YOLOv10n as the baseline for our experiments. Compared to the baseline, after introducing the improved BiFPN into the neck of the network, the B-YOLO achieved improvements of 1.5% in *mAP*@0.5, although *mAP*@0.5:0.95 decreased by 0.1%. Additionally, the number of parameters was reduced by 0.27 M, GFLOPs decreased by 0.3 G, and FPS increased from 136.4 to 144.4. The improved BiFPN’s bidirectional connections between top-down and bottom-up paths maintain fine-grained features across scales, which significantly improve small and blurred object detection accuracy. Furthermore, its weighted fusion mechanism allows the network to adaptively emphasize more informative features and suppress less relevant ones, thereby reducing confusion in scenes where objects are clustered and overlapping. Building on this, we incorporated the S_C2f into the neck. S_C2f integrates channel and spatial attention modules to dynamically adjust feature weights, enhancing the model’s focus on low-contrast regions. Furthermore, its multi-scale spatial convolutions improve the model’s adaptability to shape variations across different scales. Although BS-YOLO slightly increased the parameters and computational costs, it further enhanced *mAP*@0.5 by 0.5% and *mAP*@0.5:0.95 by 0.8% compared to B-YOLO. Finally, by integrating the EMA into both the backbone and neck, we aimed to further improve detection accuracy. The EMA uses parallel convolution branches to integrate contextual information, followed by cross-channel and spatial attention mechanisms to focus on critical features and suppress irrelevant background noise, which enhances localization accuracy for small objects and dense targets. The resultant BSE-YOLO achieved the highest *mAP*@0.5 of 83.7% and *mAP*@0.5:0.95 of 48.6%. The combination of BiFPN, S_C2f, and EMA creates a cohesive feature processing method optimized for complex underwater scenes. BiFPN’s weighted fusion mechanism integrates multi-scale features, preserving details for small and blurred objects and reducing the confusion in dense scenes. S_C2f leverages channel and spatial attention modules to emphasize features of low-contrast regions, while multi-scale spatial convolutions improve adaptability to shape and scale variations. EMA’s cross-channel and spatial attention mechanisms prioritize critical features and suppress background noise, improving localization accuracy for small and clustered objects.

When used independently, the designed S_C2f improved *mAP*@0.5 by 0.7% compared to the baseline. Subsequently, BE-YOLO, which integrates the enhanced BiFPN with the EMA, achieved further improvements of 0.2% in *mAP*@0.5 and 0.2% in *mAP*@0.5:0.95 over B-YOLO. Although the EMA module alone did not significantly boost performance, its combination with BiFPN enabled more efficient feature exchange and fusion, as well as enhanced processing of multi-scale features, thereby improving the detection accuracy. Finally, after incorporating both the EMA and the S_C2f, BSE-YOLO’s FPS decreased to 97.7, lower than the baseline. However, it still meets real-time detection requirements, striking an effective balance between efficiency and accuracy.

**Figure 9:**
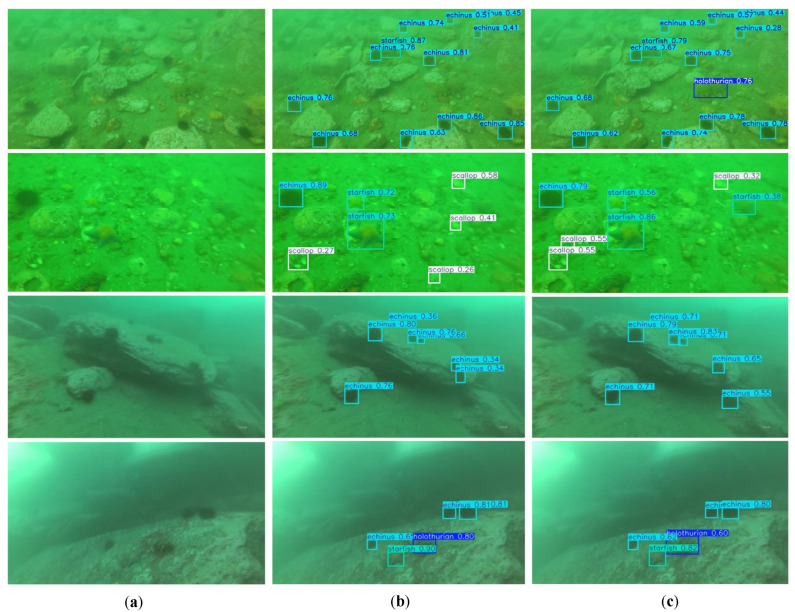
Detection results on URPC2020. (**a**) Raw image; (**b**) YOLOv10n; (**c**) BSE-YOLO.

As shown in Figure 9, to further comprehensively validate the superior performance of BSE-YOLO, we conducted qualitative testing in addition to the quantitative experiments by selecting several images from the URPC2020 test set. The results demonstrated that, compared to YOLOv10n, BSE-YOLO achieved higher accuracy in the recognition and detection of small objects, with lower probabilities of target omission and misidentification. These experimental results demonstrated that the proposed model exhibited more outstanding performance in object detection accuracy within underwater environments.

### 4.7. Application Test

**Figure 10:**
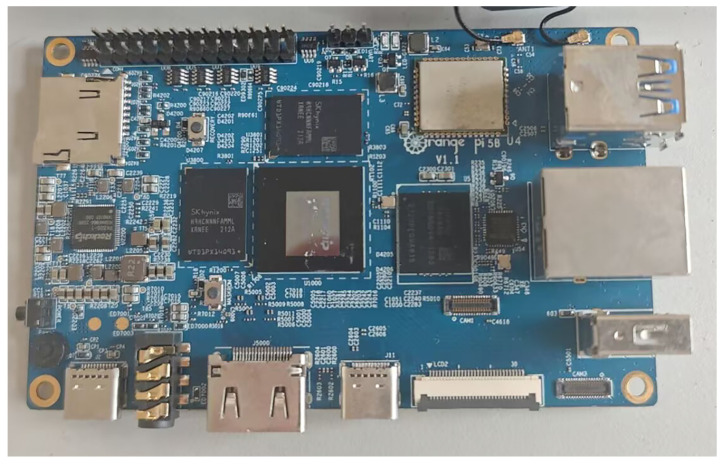
Orange Pi 5B.

We deployed our BSE-YOLO on an embedded device to assess its real-world applicability. Figure 10 displays Orange Pi 5B as the testing device, equipped with the RK3588S 8-core 64-bit processor, featuring a quad-core A76 and a quad-core A55, with a maximum frequency of 2.4 GHz. We utilized ONNX as the inference computing framework to evaluate the performance of our BSE-YOLO, with detailed information provided in Table 4. Additionally, we selected YOLOv10n for comparison. As illustrated in Table 4, we conducted tests on input images of sizes 256 × 256 and 640 × 640, respectively. Although the proposed BSE-YOLO had a slightly lower inference speed compared to YOLOv10n, it still met the detection requirements in low-computing environments.

**Figure 11:**
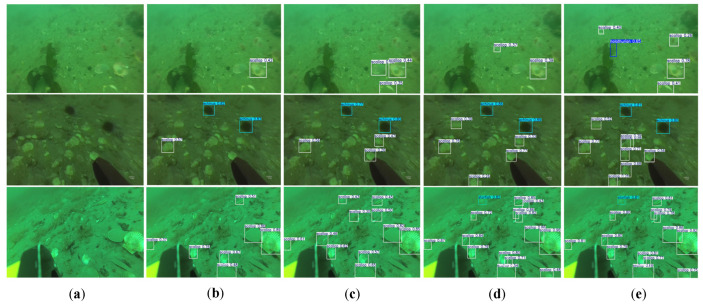
The inference results under different input resolutions. (**a**) Raw image; (**b**) YOLOv10n with 256 × 256 input; (**c**) BSE-YOLO with 256 × 256 input; (**d**) YOLOv10n with 640 × 640 input; (**e**) BSE-YOLO with 640 × 640 input.

To further demonstrate the practical detection performance of both models, we visualized the inference results under different input resolutions. As shown in Figure 11, both YOLOv10n and BSE-YOLO were capable of detecting underwater targets effectively on the Orange Pi 5B. Specifically, (b) and (d) present the detection results of YOLOv10n with input sizes of 256 × 256 and 640 × 640, respectively, while (c) and (e) display the results of BSE-YOLO under the same conditions. It was observed that BSE-YOLO achieved comparable detection accuracy with clearer target localization while maintaining acceptable inference latency on the embedded system.

## 5. Conclusions

This paper proposes a lightweight underwater object detection model, BSE-YOLO, based on YOLOv10n. To balance detection efficiency and accuracy, we introduce an enhanced BiFPN to replace the original neck and design a Multi-Scale Attention Synergy Module (MASM), which forms the core of the proposed S_C2f, which strengthens the perceptibility of challenging sample features, thereby substantially boosting overall performance. Furthermore, we integrate the EMA module into both the backbone and neck to enhance the extraction and fusion of different-scale features, further improving the recognition and detection of small objects in complex underwater environments. The experimental results demonstrate that, compared to the baseline model YOLOv10n, BSE-YOLO improves *mAP*@0.5 on the URPC2020 and DUO datasets by 2.2% and 3.0%, respectively. While reducing the network’s parameters, BSE-YOLO effectively detects small objects in complex underwater environments, achieving an excellent balance between lightweight design and high accuracy. This study primarily focuses on improving the neck of the baseline model. Future research could explore optimizing the backbone to enhance feature extraction capabilities, further improving detection accuracy, or reducing parameters to make the model even more lightweight.

## Figures and Tables

**Table 1:** Comparison of different object detection models on the URPC2020 dataset.

Methods	mAP@0.5	mAP@0.5:0.95	Parameters (M)	GFLOPs (G)	FPS
SSD [14]	76.2	37.6	24.01	274.4	70.6
YOLOv3	72.9	31.4	61.54	155.3	69.1
YOLOv4	75.4	34.0	63.95	141.9	51.9
YOLOv5s	80.4	43.5	7.27	17.1	103.1
YOLOvX [41]	79.7	42.0	5.03	15.2	102.7
YOLOv8n	82.2	47.9	3.01	8.2	140.8
YOLOv10n	81.5	47.6	2.70	8.2	136.4
FEB-YOLO [39]	83.5	48.9	1.64	6.2	-
RTDETR [42]	80.5	44.9	31.99	103.4	37.8
LUW-DETR [43]	83.1	-	14.23	40.1	-
BSE-YOLO	83.7	48.6	2.47	8.3	97.7

**Table 2:** Comparison of different object detection models on the DUO dataset.

Methods	mAP@0.5	mAP@0.5:0.95	Parameters (M)	GFLOPs (G)	FPS
SSD [14]	79.7	50.7	24.01	274.4	70.6
Faster R-CNN [17]	74.4	39.3	136.75	401.7	38.2
YOLOv3	71.6	40.3	61.54	155.3	69.1
YOLOv4	76.7	43.9	63.95	141.9	51.9
YOLOv7-Tiny	81.0	57.7	6.02	13.2	105.2
YOLOv8n	81.7	61.8	3.01	8.2	140.8
YOLOv10n	80.9	61.7	2.70	8.2	136.4
FEB-YOLO [39]	82.9	63.2	1.64	6.2	-
RT-DETR [42]	77.4	55.5	31.99	103.4	37.8
BSE-YOLO	83.9	64.2	2.47	8.3	97.7

**Table 3:** The ablation experiments on URPC2020.

Methods	mAP@0.5	mAP@0.5:0.95	Parameters (M)	GFLOPs (G)	FPS
YOLOv10n	81.5	47.6	2.70	8.2	136.4
B-YOLO	83.0	47.5	2.43	7.9	144.4
S-YOLO	82.2	47.6	2.73	8.4	103.6
E-YOLO	81.6	47.2	2.71	8.4	115.2
BS-YOLO	83.5	48.3	2.46	8.0	103.0
BE_YOLO	83.2	47.7	2.44	8.0	125.6
SE_YOLO	82.8	47.8	2.74	8.5	94.2
BSE_YOLO	83.7	48.6	2.47	8.3	97.7

YOLOv10n with BiFPN is called B-YOLO. YOLOv10n with S_C2f is called S-YOLO. YOLOv10n with EMA is called E-YOLO. YOLOv10n with BiFPN and S_C2f is called BS-YOLO. YOLOv10n with BiFPN and EMA is called BE-YOLO. YOLOv10n with S_C2f and EMA is called SE-YOLO. YOLOv10n with BiFPN, S_C2f and EMA is called BSE-YOLO.

**Table 4:** Performance of BSE-YOLO on an embedded device, Orange Pi 5B.

Methods	Size	Parameters	GFLOPs	Framework	Time (ms)
YOLOv10n	256	2.70	8.2	ONNX	44.2
BSE-YOLO	256	2.47	8.3	ONNX	57.6
YOLOv10n	640	2.70	8.2	ONNX	236.2
BSE-YOLO	640	2.47	8.3	ONNX	264.6

## Data Availability

Data will be made available on request.
